# Pen Surface Temperature as a Predictor of Daily Water Intake and Tympanic Temperature in Steers Finished in Feedlots

**DOI:** 10.3390/ani13071150

**Published:** 2023-03-25

**Authors:** Rodrigo A. Arias, Terry L. Mader

**Affiliations:** 1 Instituto de Producción Animal, Facultad de Ciencias Agrarias y Alimentarias, Universidad Austral de Chile, Valdivia 5090000, Chile; 2 Centro de Investigación de Suelos Volcánicos, Universidad Austral de Chile, Valdivia 5090000, Chile; 3 Animal Science Department, University of Nebraska-Lincoln, Lincoln, NE 68583-0908, USA

**Keywords:** thermal balance, angus steers, comfort thermal index, solar radiation, heat stress

## Abstract

**Simple Summary:**

Water scarcity is a relevant issue in a scenario of climate change not only for animal production but also for many regular human activities. Thus, an adequate estimation of its demand is of high importance due to the high requirement for animal protein worldwide forecasted for the next decades. This study highlights that pen surface temperature can be used to predict daily water intake and tympanic temperature in feedlot steers. Commercial feedlot producers can estimate their water requirements under scenarios of heat stress with a simple tool, contributing to keeping sustainable production systems.

**Abstract:**

Adequate estimation of water demand in cattle production feed yards is of high importance, especially due to reduced water availability because of changes in rain precipitation patterns and amounts. The pen surface in feed yards receives and reflects solar radiation, affecting along with other factors the microclimate to which cattle are exposed. This study aimed to describe the relationship between the pen surface temperature with the daily water intake and the tympanic temperature of finishing steers. Climate variables, including solar radiation, air temperature, relative humidity, and wind speed plus pen surface temperature and soil temperature at 10.2 cm depth were recorded. Data were collected from a weather station located in the feedlot in Concord NE, whereas daily water intake was estimated from a set of experimental pens sharing a waterer in two adjacent pens. Simple and polynomial linear regressions were assessed from data collected in different experiments conducted from 2003 to 2006. Two models to predict daily water intake were developed for finishing steers using the pen surface temperature as the predictor variable. The first one included data for the period May-October (overall model) and the second one for the summer period (June-August). The best fit for the overall model was a quadratic fit (r^2^ = 0.86), whereas the best-fit model for the summer model was the cubic (r^2^ = 0.72). Subsequently, both models were validated with data from an independent experiment conducted in the summer of 2007 in the same facilities. Both models tended to slightly overestimate daily water intake when they were validated (14.6% and 12.6%, respectively). For tympanic temperature, the best-fit model was linear, explaining 76% of the observed variability. When the dataset was split into night-time and daytime the best-fit model for the night period was a quadratic one and a linear one for the daytime, both improving the explanation of the variability observed. In conclusion, pen surface temperature can be used to predict both daily water intake and tympanic temperature in feedlot steers without access to shade.

## 1. Introduction

Water is a nutrient of recognized relevance affecting the survival and performance of cattle. Nevertheless, accessible water receives less consideration than other nutrients [1]. On the other hand, water is becoming scarce worldwide due to climate change [2] and changes in the amount and distribution of precipitation, being more frequent periods of extended droughts. Thus, an adequate estimation of water demand in cattle production is of high importance. At present, there are a limited number of studies that have addressed water intake prediction in growing and finishing cattle in feed yards [2,3,4,5,6,7].

Air temperature has been widely used as an indicator of animal comfort and performance [8,9,10]. Likewise, it is considered one of the primary factors affecting thermal balance in cattle [11]. However, along with ambient air temperature, other factors such as wind speed, humidity, and solar radiation are involved in the thermal balance of cattle [12], which in turn affects water intake behavior. Adequate estimation of environmental effects on the thermal balance of cattle requires that climatic variables be obtained at appropriate locations. For instance, air temperature decreases with height above the ground surface [13,14,15]. In addition, wind speed increases with height and depends on the roughness of the terrain and the stability of the atmosphere [16,17,18]. However, air temperature and wind speed are usually recorded at 3.0 m height, whereas the typical steer hip height is approximately 1.3 m (ranging from 1.04 to 1.52 m), with the middle of the animal estimated at around 0.9 m in height. In this regard, solar radiation has also been recognized as an important factor in animal thermal balance [9,12,19], modifying the animal body temperature as well as the respiration rate [20,21,22]. Although solar radiation has been widely studied on its direct effects on animals, less is known about the effects of it on the pen surface and the impact on the microclimate at the pens at which animals are maintained during the growing and finishing phases. Additionally, pen surface properties may change because of animal activity, precipitation, and manure deposits of organic matter that accumulate over the feeding period [23]. These changes alter the soil heat conductivity and capacity. Thus, we hypothesize that the pen surface temperature could be a reliable predictor of daily water intake and tympanic temperature in finishing steers because of the proximity of cattle to the soil and its microclimate. The objective of this study was to assess the pen surface temperature as a predictor of daily water intake on finishing cattle.

## 2. Materials and Methods

### 2.1. Micrometeorological and Water Intake Data Collection

The relationship between daily water intake (DWI), the pen surface temperature (PST), and the soil temperature at 10.2 cm depth (ST) were established using information from a set of experiments conducted from 2003 to 2006 at the Haskell Agricultural Laboratory in Concord, NE (42°23′ N latitude and 96°57′ W longitude; elevation 445 m). The pens used in this study had no shelter or windbreak. All the experiments used finishing steer Angus crossbred cattle. Climate data were collected continuously (10 min intervals) in a weather station implemented with a data logger CR10X (Campbell Scientific Inc., North Logan, Utah) and then summarized to get hourly data. The weather station was located at the center of the fence line dividing the two central pens of the alley. Soil temperature (10.2 cm depth) was recorded using a thermistor model 107 (Campbell Scientific Inc., North Logan, Utah), which was located within a tube buried next to the fence that separated pens and attached to the weather station. Pen surface temperature was recorded using a laser infrared gun located approximately 2.0 m above ground. The laser gun was attached to the weather station and directed to the center of the mound in the center of the pen (approximately 1.5 m from the fence) an area commonly used by the animals. Wind speed was recorded using an anemometer model 014A (Met One Instruments, Inc., Grants Pass, Oregon), whereas air temperature and relative humidity were recorded with an HMP35 sensor (Campbell Scientific Inc., North Logan, UT, USA). Net solar radiation was obtained from the High Plains Climate Center automated weather station located 0.6 km west and 1.5 km north of the feedlot near Concord, NE. Daily water intake was estimated by dividing the total water intake by the number of animals in each set of two adjacent pens (*n* = 17), which shared a common water tank.

In the summer of 2007 (26 June to 15 August 2007), an experiment was conducted to validate the equations obtained to predict daily water intake (DWI). In this experiment, a total of 112 Angus crossbred steers (7 heads/pen, mean BW 417 ± 3.4 kg) were fed a finishing diet based on dry-rolled corn (76% DM). Variables of interest were collected over a 51-day period. In addition, two steers per pen were fitted with a data logger iButton^®^ (DS1922L, Nexsens Technology, Beavercreek, OH, USA) to collect tympanic temperature (TT) as an estimator of core body temperature. Hourly TT were collected over a 7-day period, from 5 to 12 July 2007. These data were also used to assess the relationship between PST, ST, and air temperature (AT). In addition, for this period the day was divided into daytime (0700 to 2000) and nighttime (2100 to 0600). Finally, simple linear, quadratic, and cubic regression models were estimated using net solar radiation to predict DWI.

### 2.2. Statistical Analysis

The data set used to obtain the DWI prediction equations was divided into two groups: (1) the overall model representing the period May to October, and (2) the summer model representing the period June to August. The data were analyzed using statistical packages JMP^®^ (Version 5.0.1.2, SAS Inc., Cary, NC, USA) and SAS^®^ (Version 9.01, SAS Inc., Cary, NC, USA). Scatterplots and ANOVA were used to assess the relationship and differences among ambient AT, PST, ST, and black-globe temperature. Simple linear and polynomial regression analyses were conducted to obtain DWI equations based on ST and PST. Finally, the models were assessed using a graphical representation of actual DWI, predicted DWI, and the analysis of the residuals of each model using data collected during the summer of 2007. For the hourly TT prediction model data from PST, ST, and TT from the experiment conducted in Concord, NE was used. All data collected during the 7 days period (climatic and TT) were averaged per hour of the day and then modeled. One model was obtained for the overall period 24 h dataset, then data was split into two subsets one for the night-time from 2100 to 0600 h and another for daytime from 0700 to 2000 h. Correlations and linear, quadratic, and cubic linear regressions were assessed in each case.

## 3. Results

### 3.1. Daily Water Intake Model Development

**Figure 1:**
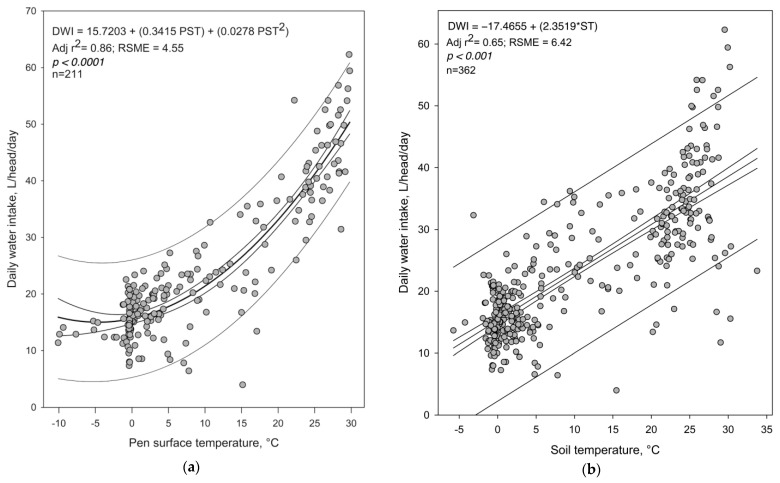
Relationship of daily water intake (DWI) and pen surface temperature (PST) with best fitting equations for (**a**) the overall model (May to October) and for (**b**) the summer model (June to August.

Regression equations (linear and polynomial) were obtained using both data sets (overall period and summer season) and compared to determine which variables in the study best explained DWI in finishing cattle. The mean DWI was 23.56 ± 0.57 and 34.15 ± 0.76 L/head/day for the overall and summer season periods, respectively. Summer DWI was similar to those reported by Hicks et al. [4] for the period of July-August, Jeter [24] for a 150-day feeding period in Texas, and Arias and Mader [5] in Nebraska. Table 1 shows a summary of the adjusted r^2^ (Adj. r^2^) for ST and PST. Pen surface temperature was a better predictor of DWI than ST for the summer and overall periods (Adj. r^2^ = 0.69 vs. 0.08 and 0.82 vs. 0.65 for the summer and overall periods, respectively). Figure 1 displays the fits of the summer and overall models, as well as their respective equations using the PST as the predicting variable. Figure 1a shows a positive response of the amount of DWI to PST (*p* < 0.01). The best fit for the overall model was a quadratic one (Adj. r^2^ = 0.86, Table 1, *p* < 0.01). The cubic model only improves in 1% the observed variability regarding the quadratic one. However, in the summer the cubic polynomial equation increased from 0.69 to 0.72 (Table 1). Figure 1b shows the linear relationship between ST and DWI during the study period (overall and summer models). In the overall model, a steer increases its DWI by 2.4 L per degree increase in ST, between −5 and 35 °C (*p* < 0.01). The quadratic and cubic polynomial models did not improve the Adj. r^2^. In the summer, although the linear and quadratic models were significant, they explain poorly the variability observed in DWI (Adj. r^2^ < 0.08).

The overall model corresponds to the May-October period, whereas the summer model includes the June-August period. Soil temperature was recorded at 10.2 cm depth next to the fence dividing the central pen. Pen surface temperature was recorded with a laser gun located at approximately 2.0 m above ground and directed to the mound in the center of the pen, an area commonly used by the animals.

### 3.2. Tympanic Temperature Model Development

**Figure 2:**
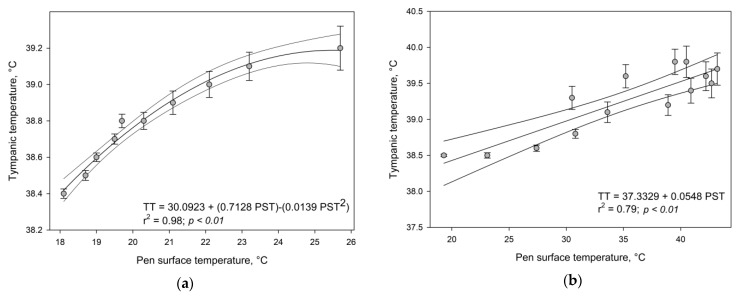
The best fitting equations for predicting tympanic temperature (TT) using pen surface temperature (PST) as a predictor variable (**a**) = nighttime from 2100 to 0600 h, and (**b**) for daytime from 0700 to 2000 h).

The mean TT and mean PST, for the period of seven days of validation, are presented in Table 2. Pen surface temperature, as expected, was higher during the daytime and lower during nighttime (34.8 ± 2.05 vs. 20.7 ± 0.74 °C, respectively; *p* < 0.001). Similarly, TT was also higher in the daytime than during nighttime (39.3 ± 0.13 vs. 38.8 ± 0.08 °C, respectively; *p* = 0.008). The best-fit model for the 24 h period was linear explaining about 75% of the observed variability (TT = 37.8331 + 0.0423 PST, r^2^ = 0.7581). Meanwhile, the quadratic and cubic models were non-significant (*p* > 0.05). When the dataset was split into night-time and daytime the best-fit model for the night period was a quadratic one and a linear one for the daytime (Figure 2). The correlation values for TT and PST night and daytime were 0.92 and 0.89, respectively. The daytime model was non-significant (*p* > 0.05) for quadratic and cubic polynomials.

**Figure 3:**
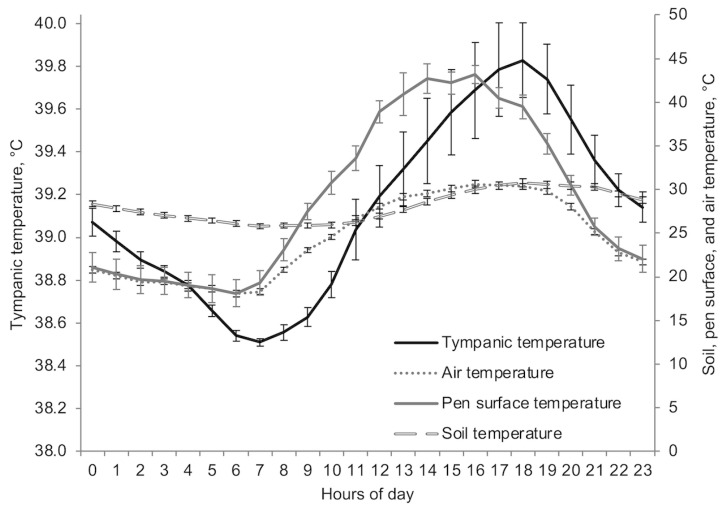
The least mean squares of soil temperature (at 10.2 cm depth), pen surface temperature, air temperature, and tympanic temperature by the hour of the day (5 to 12 July 2007). The bars within each variable correspond to the respective standard error of the mean.

Figure 3 displays the average hourly values of ST, PST, AT, and TT from 5 to 12 July 2007. Soil temperatures had the lowest variation throughout the day and were higher than those recorded for AT during the evening and night-time but were lower than the PST during the daytime. Air temperature and ST showed a pattern similar to TT with an increase during the daytime and a decrease during the night-time, showing changes within 24 h period (*p* < 0.0001). Soil temperature was higher than air temperature between 2000 and 0900 h, whereas no differences were found between 1000 and 1900 h (*p* > 0.05). Likewise, PST was similar to AT between 2100 and 0700 h (*p* > 0.05), and it had a similar pattern to net solar radiation since its values quickly increased after sunrise reaching its peak between 1300 and 1800 h (net solar radiation data not shown). For the period of study (5 to 12 July 2007), the mean PST was 4.6 and 0.93 °C greater than the mean ST and the mean AT, respectively (29.0 ± 1.89, 28.0 ± 0.37, and 24.4 ± 0.97, *p* < 0.0001), whereas the daily mean TT was 39.1 ± 0.094 °C.

### 3.3. Daily Water Intake Models’ Validation

Table 2 summarizes the main climatic variables, the temperature-humidity index, and TT for the experiment conducted at Concord, NE (5 to 12 July 2007) to validate the models. Although mean values for the PST and AT were similar, there was a great variation across the day (Figure 3). On the other hand, ST had a higher mean value but less variation across the day (range = 8.84). During the experimental period mean wind speed showed a bell pattern with higher values after 0900 h (>2.7 m/s), reaching the maximum values between 1200 and 1800 h (>3.0 m/s) and the peak of 3.4 m/s at 1500 h. After that, mean wind speed decreases quickly to values lower than 1.4 m/s at 2100 h. During the nighttime, values ranged from 1.0 to 1.4 m/s. In this manner, the time of day that had the maximum wind speed coincided with the time of day with maximum solar radiation, helping cattle to alleviate heat load.

**Figure 4:**
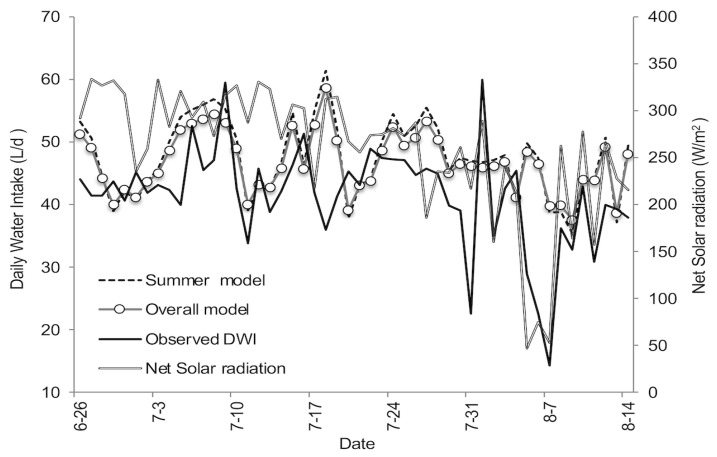
Observed and predicted daily water intake (DWI) using the pen surface temperature as the predictor variable in both models (overall and summer) for the period 27 June to 15 August 2007.

The observed and predicted DWI are presented in Table 3. The mean DWI recorded was higher than that reported by other researchers [3,6,25] and similar to previous reports by Hicks et al. [4], Jeter [24], and Arias and Mader [5]. Observed DWI and net solar radiation of the validation experimental period are shown in Figure 4, along with predicted DWI using the PST as the predictor variable (summer and overall models). For instance, the peak of DWI of 59.9 L/day was observed on 2 August 2007, whereas the lowest DWI recorded was on 8 August 2007 (14.3 L/day). This drop in DWI appears to be related to the drop in net solar radiation. However, when simple and quadratic linear regression models were performed using net solar radiation with the validation dataset the Adj. r^2^ were 0.42 and 0.47, respectively (*p* < 0.01). The relationship between net solar radiation and DWI was clear during August but was nonexistent during July (Table 4).

In general, models tended to slightly overestimate DWI by 14.6% and 12.6% for the summer and overall models, respectively. Despite the difference in the mean DWI, the differences between the model predictions and the observed values were similar (2.8% and −2.1% for the summer and overall models, respectively). Both models overestimated the minimum DWI (148% and 163% for the summer and overall periods, respectively). The residuals (deviations) of both models ranged from −14.03 to 25.5 and the sum of the squares of the residuals was 5419 for the summer model and 4618 for the overall model.

## 4. Discussion

At present, a limited number of studies have tried to predict DWI in growing cattle finished in feed yards [3,5,6,7,26]. Most of the equations obtained in those studies used climate and animal variables as predictor factors as it was summarized by Ahlberg et al. [7]. Thus, the present work seems to be the first study reporting the relationship between PST on cattle DWI. In fact, only a limited number of studies have reported pen (ground) surface temperature and its relationship with hide temperature [27], animal performance [28,29,30], the chemical composition of pen surface [23], and greenhouse gas emissions [31].

Previous studies conducted in Nebraska by [15,32] have shown that sprinkling feedlot cattle during heat waves modifies the microclimate to which animals are exposed, resulting in a reduction of heat stress incidence. In their experiment, Mader et al. [15] sprinkled cattle for 20 min at intervals of 1.5 h during the morning and the afternoon when the maximum temperature-humidity index (THI) for the day was predicted to be ≥ 77. The authors reported that although relative humidity was increased, THI values were lower for treatments where cattle were sprinkled. Wetting cattle and soil at the same time decreased ST and air temperature at 1.0 m above ground in different magnitudes. These differences were greater after noon and averaged 5.2 °C and 1.4 °C, respectively. In addition, cattle tended to occupy the areas where sprinkling took place, even during days when sprinkling had not occurred. A similar response was reported by Marcillac-Embertson et al. [33] with heifers spending more time in the wet areas of the pen. In the Southern California desert region, Carvalho et al. [30] reported that the temperature of dry pen surfaces was greater than that of muddy surfaces (50.3 vs. 20.4 °C, respectively) during the summer months. Thus, PST has a direct effect on the microclimate, impacting cattle behavior [15]. It must be noticed that water that is evaporated from the pen surface reduces air temperature immediately above the ground because energy is used to evaporate the water from the soil [34,35]. Although water has a thermal conductivity of about 25 times greater than air, it takes over 3000 times as much energy to warm an equal volume of water as compared to air [35]. Similarly, water reduces the body temperature of the animal with a wet hair coat. Because of wetting both the soil and the animal’s body, there is an increase in the heat gradient. This allows a greater heat flow away from the animal through conduction and convection processes. On wet soils, the latent heat flux consumes most of the energy from net radiation. Hence, when a pen surface is wetted, there is less energy available for heating the soil and the animal. In addition, less radiant energy will be released from the pen surface because of a decrease in ST, which drives long-wave radiation emissions. Thus, these changes in soils and pen surface conditions from dry to wet could also explain the water-drinking behavior of animals. However, as the pen surface dries and water becomes less available for evaporation, the energy must go into soil heat flux or sensible heat flux [36].

Several studies have demonstrated that cattle increase water intake during summertime [5,7,37,38]. In addition, previous studies have shown that mean air temperature, as well as the maximum and minimum air temperatures, solar radiation, wind speed, and/or THI are able to explain most of the variability in water intake, either during the summer or through the year [24,26]. Arias and Mader [5] reported that seasonal simple regression equations for the summer model produced low Adj. r^2^ values for all the environmental variables studied (r^2^ < 0.15). However, these Adj. r^2^ values were improved when data from both seasons were pooled (minimum temperature Adj. r^2^ = 0.57, maximum temperature Adj. r^2^ = 0.52, and solar radiation Adj. r^2^ = 0.50). The authors also reported that a multiple regression analysis of pooled data had an R^2^ of 0.71 for a model including minimum temperature, solar radiation, and dry matter intake. When THI was used in the model instead of the minimum temperature it produced a similar R^2^. For the data presented herein, r^2^ values of 0.86 and 0.70 were obtained for the overall and the summer models, respectively. The values herein reported were similar to those reported by Arias and Mader [5], resulting in a simpler and better equation to estimate DWI. Although the models perform well in estimating the mean and maximum DWI, they failed in estimating minimum DWI. This lack of precision indicates that other factors may be influencing DWI by cattle. Therefore, we recommend further studies exploring the relationship between solar radiation and PST as predicting factors of DWI.

Regarding the TT model, the results herein presented could be a promising tool to indirectly assess the thermal comfort of animals in feed yard conditions during summertime. The increase in the PST and TT observed in the daytime can be explained because of the higher net solar radiation that pens and animals are exposed to in the daytime. In this case, averaged 276.2 W/m^2^, whereas in the nighttime it was −39.7 W/m^2^, which means that the pen surface soil lost energy into the atmosphere. Likewise, the great variation of solar radiation can also explain the greater variability observed for both in the day (0.13 vs. 0.08 and 2.05 vs. 0.74 for TT and PST in day and night respectively). The night model explains most of the TT observed at 98%, whereas the day model only explains 79%, meaning that during the daytime other factors can be contributing to TT and consequently to the thermal balance of the animal.

Arias and Mader [39], reported that wind speed and outgoing longwave solar radiation were the best predictors of TT of animals. The authors pointed out that longwave solar radiation explained 73.7% of the variability in TT, whereas wind speed explained 4.9%. They also showed a high relationship between outgoing longwave solar radiation and PST. This can be explained because the earth and atmospheres are major sources and sinks of longwave radiation. Altogether our results demonstrate the relevance of net solar radiation on DWI and the animal thermal balance.

## 5. Conclusions

The pen surface temperature affects both daily water intake and tympanic temperature in finishing steers being a reliable predictor of both. Further studies considering other variables affecting solar radiation absorption and emission by soil are needed to understand the relationship with the animal response. These factors should include soil textures, degree of soil compaction, organic matter content, and pen surface temperature variability within the pen and between pens either, because these factors can vary across feed yards.

## Figures and Tables

**Table 1:** Adjusted coefficients of determination (*p*-values) for models’ prediction of daily water intake (DWI) using soil temperature and pen surface temperature.

Predictor Variable and Models		Linear	Quadratic	Cubic
Soil temperature	Overall *n* = 362	0.65 (<0.01)	0.65 (0.02)	0.65 (0.28)
	Summer *n* = 143	0.08 (<0.01)	0.11 (0.08)	0.10 (0.64)
Pen surface temperature	Overall *n* = 211	0.82 (<0.01)	0.86 (<0.01)	0.87 (<0.01)
	Summer *n* = 55	0.69 (<0.01)	0.68 (0.85)	0.72 (<0.01)

**Table 2:** Summary of main climatic variables and tympanic temperature for the week of validation (5 to 12 July 2007).

	ST, °C	AT, °C	PST, °C	SR, W/m^2^	WS, m/s	THI	TT, °C
Mean	27.98	24.22	24.41	197.50	2.31	71.43	39.24
SEM	0.38	0.91	1.50	43.50	0.21	1.16	0.08
Min	23.31	11.50	7.74	−80.83	0.61	52.96	38.52
Max	32.15	34.46	40.82	683.32	6.00	83.25	40.44

Abbreviation: ST = Soil temperature at 10.2 cm depth; AT = Air temperature; PST = Pen surface temperature; SR = Net solar radiation; TT = Tympanic temperature; THI = Temperature-humidity index; WS = Wind speed. SEM = Standard error of the mean.

**Table 3:** Summary for daily water intake (DWI) for the 51-day evaluation period in Concord NE, 26 June to 15 August 2007 (L/head/d).

Item	Observed Daily Water Intake	Predicted Daily Water Intake	
		Summer Model	Overall Model
Mean	41.5	47.6	46.7
SEM	1.1	0.9	0.7
Maximum	59.9	61.4	58.7
Minimum	14.3	35.4	37.5
Range	45.6	26.0	21.2

Summer model, DWI = −17.4655 + (2.3519 × PST); Overall model, DWI = 15.7203 + (0.3415 × PST) + (0.02878 × PST^2^); where PST = Pen surface temperature (°C).

**Table 4:** Adjusted coefficients of determination for regression equations and correlation values for observed daily water intake and net solar radiation in finishing steers with the validation dataset.

Model and Correlation	Overall Period	July (31 Days)	August (15 Days)
Linear	0.42	ns	0.56
Quadratic	0.47	ns	0.55
Cubic	0.48	ns	0.54
Correlation	0.66	0.02	0.77

The whole period corresponds to 50 days (from 27 June to 15 August 2007). Only significant coefficients are shown (*p* < 0.01), otherwise ns is shown (ns = not significant).

## Data Availability

The data that support the study findings are available from the authors upon request and after authorization by all authors.
